# Development of dynamical weather-disease models to project and forecast malaria in Africa

**DOI:** 10.1186/1475-2875-11-S1-P133

**Published:** 2012-11-09

**Authors:** Volker Ermert, Andreas H Fink, Andrew P Morse, Anne E Jones, Heiko Paeth, Francesca Di Giuseppe, Adrian M Tompkins

**Affiliations:** 1 Institute of Geophysics and Meteorology, University of Cologne, Cologne, Germany; 2 School of Environmental Sciences, University of Liverpool, Liverpool, UK; 3 Institute of Geography, University of Würzburg, Würzburg, Germany; 4 European Centre for Medium-range Weather Forecasts (ECMWF), Reading, UK; 5 Earth System Physics, Abdus Salam International Centre for Theoretical Physics (ICTP), Trieste, Italy

## Background

Weather and climate play an important role in the spread of malaria. Suitable weather conditions for malaria are found in sub-Saharan Africa, where most of the worldwide malaria cases and deaths are found. For this reason, integrated weather-disease malaria models are useful tools to project the malaria future and to provide monthly-to-seasonal forecasts.

## Methods

Malaria projections and forecasts are undertaken by two dynamical mathematical-biological malaria models: (i) the LMM (Liverpool Malaria Model) [1-3] and (ii) VECTRI (VECtor-borne disease community model of the International Centre for Theoretical Physics, TRIeste). Both models are driven by daily temperature and precipitation values. An improved version of the LMM was introduced by [2], which was calibrated by malaria field observations from West Africa [3]. Regarding the assessment of the impact of climate change on malaria [4], the LMM was driven by data from the REgional MOdel (REMO) including the effect of land surface changes.

For the QWeCI (Quantifying Weather and Climate Impacts on health in developing countries) project, a seamless weather prediction system has been developed at ECMWF by appending the first 25 days of the monthly forecasting system with the Seasonal Forecasting System 4 to provide a continuous 120 day lead time prediction. The forecast is calibrated to correct for displacement errors of West African monsoonal precipitation.

## Results and outlook

The malaria projections up to 2050 [4] based on the integrated REMO-LMM reveal a southward shift of the epidemic malaria area in West Africa due to the precipitation decline. The increased temperatures lead to an increase of transmission in highland territories. Formerly, malaria free areas become epidemic, whereas the epidemic risk is decreased in lower-altitude regions.

Actual research within the EU Seventh Framework Programme (FP7) QWeCI and HEALTHY FUTURES projects is underway to exploit the feasibility of monthly-to-seasonal malaria forecasts. QWeCI is currently developing prototype seamless malaria forecasts for Malawi (http://nwmstest.ecmwf.int/products/forecasts/d/inspect/catalog/research/qweci/).

The LMM and VECTRI neglect various important malaria factors like immunity, malaria control activities, or different vector characteristics. Further development of VECTRI will be undertaken to include other relevant malaria factors. Note that VECTRI represents a community model meaning that the model and code is publicly available (http://users.ictp.it/~tompkins/vectri/). The LMM is included in the so-called Disease Model Cradle (DMC) that is downloadable (http://www.liv.ac.uk/qweci/project_outputs/). Open-access web versions of both models are applicable for point data (see http://qweci.uni-koeln.de).
